# Gender and Hemispheric Asymmetries in Acquired Sociopathy

**DOI:** 10.3389/fpsyg.2019.00346

**Published:** 2019-03-19

**Authors:** Ricardo de Oliveira-Souza, Thiago Paranhos, Jorge Moll, Jordan Grafman

**Affiliations:** ^1^Department of Neurology and Neuropsychiatry, D'Or Institute for Research and Education, Rio de Janeiro, Brazil; ^2^Departments of Neurology and Psychiatry, Federal University of the State of Rio de Janeiro, Rio de Janeiro, Brazil; ^3^School of Medicine, Federal University of the State of Rio de Janeiro, Rio de Janeiro, Brazil; ^4^Shirley Ryan AbilityLab, Department of Physical Medicine, Rehabilitation, and Psychology, Neurology, Cognitive Neurology and Alzheimer's Center, Feinberg School of Medicine, Weinberg College of Arts and Sciences, Northwestern University, Chicago, IL, United States

**Keywords:** acquired sociopathy, frontal lobe syndromes, hemispheric asymmetry, morality, orbitofrontal syndrome, psychopathy, ventromedial prefrontal cortex

## Abstract

The emergence of enduring antisocial personality changes in previously normal individuals, or “acquired sociopathy,” has consistently been reported in patients with bilateral injuries of the ventromedial prefrontal cortex. Over the past three decades, cases of acquired sociopathy with (a) bilateral or (b) unilateral sparing of the ventromedial prefrontal cortex have been reported. These cases indicate that at least in a few individuals (a') neural structures beyond the ventromedial prefrontal cortex are also critical for normal social behavior, and (b') the neural underpinnings of social cognition may be lateralized to one cerebral hemisphere. Moreover, researchers have presented evidence that lesion laterality and gender may interact in the production of acquired sociopathy. In the present review, we carried out a comprehensive literature survey seeking possible interactions between gender and hemispheric asymmetry in acquired sociopathy. We found 85 cases of acquired sociopathy due to bilateral (*N* = 48) and unilateral (*N* = 37) hemispheric injuries. A significant association between acquired sociopathy and right hemisphere damage was found in men, whereas lesions were bilateral in most women with acquired sociopathy. The present survey shows that: (i) the number of well-documented single-cases of acquired sociopathy is surprisingly small given the length of the historical record; (ii) acquired sociopathy was significantly more frequent in men after an injury of the right or of both cerebral hemispheres; and (iii) in most women who developed acquired sociopathy the injuries affected both cerebral hemispheres. These findings may be especially valuable to neuroscientists and to functional neurosurgeons in particular for the planning of tumor resections as well as for the choice of the best targets for therapeutic neuromodulation.

## Introduction: Acquired Sociopathy and Frontotemporoinsular Damage

Eslinger and Damasio coined the expression “acquired sociopathy” to describe the changes in personality of evr, a comptroller in a home-building firm who underwent a lasting change in personality following the surgical removal of an olfactory groove meningioma. evr's normal cognitive performance contrasted with his severe loss of social tact and comportment, which culminated in bankruptcy and abandonment by his wife and friends. In contrast to individuals with developmental psychopathy, who “never learn socially acceptable patterns of behavior” (Eslinger and Damasio, 1985, p. 1737), evr had learned and engaged in such patterns for most of his life; however, after his brain damage he failed to behave accordingly in real-life situations. The report by Eslinger and Damasio gave breath to the study of the neural underpinnings of human social behavior and its drastic changes following damage to the vmPFC (Barrash et al., 2000).

Pari passu with the renewed interest in the antisocial changes of personality due to vmPFC damage, investigators have documented the emergence of acquired sociopathy in lesions outside the vmPFC, more specifically, in dorsolateral prefrontal cortex (Eslinger et al., 1999; Eslinger and Biddle, 2000), rostrobasal forebrain (Poeck and Pilleri, 1965; Gorman and Cummings, 1992), ventromedial hypothalamus (Flynn et al., 1988), anterior temporal lobes (Relkin et al., 1996; Miller et al., 1999), anterior cingulate (Tow and Whitty, 1953; Angelini et al., 1980), medial thalamus (Sandson et al., 1991), basal ganglia (Richfield et al., 1987; Mendez et al., 1989), and rostral brainstem (Omar et al., 2007). In most cases, the behavioral changes were enmeshed in a fabric of more elementary symptoms, such as somnolence and hyperphagia, which usually reflect damage to functionally heterogeneous neural systems (Alpers, 1937). Conversely, sociopathy was surprisingly absent in patients with even extensive bilateral prefrontal (PF) damage (Penfield and Evans, 1935; Rylander, 1939; Hebb and Penfield, 1940; Nichols and Hunt, 1940; Ghosh et al., 2014; Plaza et al., 2014); still in others the brain damage had little if any impact on socio-occupational status (Tranel et al., 2005), exerting even a paradoxically beneficial effect in some (e.g., Labbate et al., 1997; cases of and eb, Storey, 1970; King et al., 2017). These discrepancies raise the possibility that in at least some cases the laterality of the hemispheric lesion may be decisive for the development of sociopathy; in other words, damage to one cerebral hemisphere might be sufficient to produce sociopathy in at least a few previously normal individuals. A reliable prediction of whom these individuals are might bear important theoretical and practical implications.

Despite the aforementioned clues on a possible asymmetric representation of acquired sociopathy in the cerebral hemispheres, few researchers have pursued this line of inquiry. The most consistent studies on lesion laterality and acquired sociopathy have been carried out by Tranel and colleagues, who additionally investigated a possible interaction between lesion laterality and gender. In four thoroughly matched cases, they found that acquired sociopathy in one man resulted from damage to the right vmPFC, while in one woman it was caused by damage to the left vmPFC; the reverse match was not attended by changes in personality (Tranel et al., 2005). These findings were later extended to four patients suffering from pharmacoresistant epilepsy who underwent unilateral ablation of the right or the left anterior temporal lobe. The anterior temporal lobes encompass a collection of neural structures which sustain profuse bidirectional connections with the ventromedial, orbitofrontal and insular cortices as well as with the amygdala (Pascalau et al., 2018). As in the vmPFC cases, sociopathy was associated with left lobectomy in one woman and with right lobectomy in one man (Tranel and Hyman, 1990). Thus, besides lesion laterality, the Tranel et al. studies imply that gender should likewise be considered critical for the production of acquired sociopathy (Tranel and Bechara, 2010).

Gender asymmetry has been supported by studies which have consistently found higher rates of antisocial behavior among men relative to women. This “gender gap,” which is already noticeable at an early age, remains stable from childhood to adulthood (with the exception of a discrete period limited to adolescence), and has been documented in different cultures (Choy et al., 2017). Although the male-to-female ratio of lifelong antisocial conduct is 10:1, research has also shown that boys and girls with persistent antisocial behavior are identical in terms of poor discipline, family adversity, pattern of cognitive deficits, undercontrolled temperament, hyperactivity, and rejection by peers; the relaxation of diagnostic criteria of conduct disorder for girls did not substantially change these conclusions (Moffitt et al., 2004). Therefore, the weight of the evidence indicates that men are referred more often than women to psychiatric treatment and to the justice system because of differences that are intrinsic to gender, the cultural milieu playing an adjunctive role by either enhancing or curbing the overt expression of antisocial acts.

A growing body of neuroscientific research has otherwise shown that the aforementioned differences largely reflect the aspects of the cerebral organization that underpin differences in men and women (Cahill, 2006). A representative instance of the interplay between gender biology and culture was provided by Fumagalli et al. (2010), who studied the performance of 100 right-handed adults (50 men and 50 women) on a moral judgment task; volunteers were further sorted into Catholics and non-Catholics according to their stated religious belief systems. They found that only gender predicted the kind of moral judgements by men and women, men producing a significantly higher proportion of utilitarian responses than women; religious belief and education played no role in differentiating the styles of moral decisions of men and women. The authors concluded that cultural factors exerted little, if any, influence on moral judgments, suggesting that the significant factor was gender-specific differences in neural organization. Functional neuroimaging studies concur with this view. For example, in a study on the cerebral correlates of judgments of procedural justice, the overall volume of cerebral activations in women were asymmetrical (total volume of activations = 7,449 mm3) with a leftward preponderance (left hemisphere activations exceeding right hemisphere activations = 1,698 mm3); activations in men were also asymmetric (total volume of activations = 10,082 mm3), but with a clear rightward preponderance (right hemisphere activations exceeding left hemisphere activations = 5,334 mm3); qualitatively similar results were obtained for judgments of distributive justice (Dulebohn et al., 2016). Recently, the ingenious application of neuroimaging methods to criminal cases with focal brain damage collected from the literature has been done with remarkable success (Darby et al., 2018). In this study, Darby et al. did not investigate if the neural networks that they found to underpin criminal behavior differed between men and women or between the cerebral hemispheres.

To test the gender/laterality interaction in acquired sociopathy we searched the literature for *clinically*-defined cases in which an enduring antisocial change in personality had been caused by a circumscribed, non-degenerative, brain lesion. Our main goals were to investigate (i) whether unilateral hemispheric lesions are sufficient to cause acquired sociopathy, and (ii) whether gender and lesion laterality interact to modulate the final emergence of acquired sociopathy.

## Materials and Methods

### Inclusion and Exclusion Criteria

An extensive literature search was conducted using the electronic databases PubMed, ISI Web of Science, and PsycInfo to identify articles published until April 2018. We used the following search terms in title, abstract or keywords: (“sociopath^*^” or “psychopathy” or “acquired” or “frontal lobe” or “damag^*^” or “injur^*^”) and (“imaging” or “magnetic resonance imaging” or “computed tomography” or “positron emission tomography” or “brain”). Additional papers were retrieved by checking the literature cited in the articles identified by the electronic database search as well as from the authors' personal archives. We retained all single cases or case series of previously normal children, adolescents or adults who developed a persistent antisocial change in personality following an injury of the frontal lobes with or without extension into the insula, temporal pole, and neighboring subcortical regions. The reason for extending the anatomical boundaries beyond the frontal lobes is the fact that patients with sole or predominantly temporopolar injury may also present severe antisocial personality changes (Miller et al., 1999). Note that the cases included in the present survey were selected solely on the basis of the *behavioral* criterion of persistent antisocial changes in personality, not on *anatomical* criteria such as lesion location or type. By definition, cases of developmental psychopathy fell outside the scope of the present review.

For the purposes of the present study, sociopathy was operationally defined as (*a*) a chronic, recurrent and pervasive pattern of disregard for, and violation of, the rights of others (American Psychiatric Association, 2000), which (*b*) broadly overlaps Factor 2, but not necessarily Factor 1, of the Hare Psychopathy Checklist (Hart et al., 1995). As extensively discussed elsewhere, virtually all individuals with a diagnosis of psychopathy meet a diagnosis of antisocial personality disorder, or sociopathy, but the converse is not necessarily true (Lykken, 2018). However, no attempt was made in this review to differentiate sociopathy from psychopathy sensu stricto because only recently have researchers begun to differentiate acquired from developmental psychopathy (Moll et al., 2003; Mitchell et al., 2006a). With a few notable exceptions (Karpman, 1947), the earlier authors used the terms sociopathy and psychopathy interchangeably, thus overlooking the essential personality traits that distinguish psychopathy from the sociopathic behavior of other neuropsychiatric disorders (Koenigs and Tranel, 2006). We were also careful to exclude patients with antisocial changes in personality secondary to a mood disorder (Thorneloe and Crews, 1981; Bakchine et al., 1989; Tyrer and Brittlebank, 1993) and dementia (Mendez et al., 2011). In the absence of formal measures of intelligence, evidence that overt dementia or mental retardation were not part of the clinical picture were obtained from the report, otherwise the case was excluded from further analysis. A history of seizures or loss of consciousness were not a reason for exclusion, but patients with delusions, hallucinations, or both (Malloy and Richardson, 1994; Harrington et al., 1997) as well as cases with solely deep subcortical (e.g., Richfield et al., 1987; Mendez et al., 1989), or brainstem (e.g., Omar et al., 2007) lesions were excluded. Cases of sociopathy following psychosurgery were allowed in if they met the preceding criteria. Only cases in which a causal nexus between the cerebral damage and acquired sociopathy could be inferred were selected. A causal nexus was primarily provided by evidence of (i) a normal premorbid personality, and (ii) a temporal relationship between the cerebral injury and the advent of the antisocial personality change (Vann, 1972). For our purposes, “normal” was operationally defined as the capacity to be productive in the chief domains of social and interpersonal life, especially in those niches represented by family, parental/marital, school/work, and extrafamilial life (Endicott et al., 1976). Due to the considerable interindividual variation in cytoarchitectonics (Zilles and Amunts, 2012) and the less than optimal neuroanatomical accuracy of several reports prior to the 1980's, no attempt was made at this time to chart the lesions on standard cytoarchitectonic maps; for similar reasons, we did not attempt to infer the topography of the cerebral lesions based on cranial landmarks as was usual before the advent of modern neuroimaging (e.g., Jarvie, 1954). In view of the fragmentary and heterogeneous nature of the available material, no attempt was made either to sort the personality changes into subordinate domains (Barrash et al., 2018) or to perform a formal meta-analysis. Finally, we accepted as valid four cases of vmPFC dysplasia (Table 1: cases U-8 and U-10; Table 2: cases B-32 and B-47) and one case of orbital pachygyria (Table 2: case B-15). Although not strictly “acquired”, we retained these cases because the lesions in each were circumscribed to frontotemporal regions known to produce acquired sociopathy in typical cases. A similar line of reasoning has been followed by other authors (Trebuchon et al., 2013).

**Table 1:** Acquired sociopathy due to Unilateral (U) hemispheric damage (mania, mental retardation, and dementia excluded).

**Code**	**Side^a^**	**Identification**	**Sex^b^**	**Age at injury^c^**	**Handedness^d^**	**References**
*U-1*	LH	NR	M	NR	NR	Sullivan, 1911
*U-2*	LH	Case 5	M	48	R	Miller et al., 1986
*U-3*	LH	JZ	M	33	R	Meyers et al., 1992
*U-4*	LH	DT/D1	W	7	R	Eslinger et al., 1992; Anderson et al., 2006
*U-5*	LH	Mr. A/Spyder Cystkopf	M	congenital or early infancy	R	Paradis et al., 1994; Relkin et al., 1996
*U-6*	LH	BM	M	congenital	R	Fine et al., 2001
*U-7*	LH	2748	W	30	R	Tranel et al., 2005
*U-8*	LH	DK	M	26	R	Mitchell et al., 2006b
*U-9*	LH	3310	W	49	R	Tranel and Bechara, 2009
*U-10*	LH	BW	M	congenital	R	Boes et al., 2011
*U-11*	LH	NR	M	48	NR	Gilbert and Vranič, 2015
*U-12*	RH	HF	M	53	NR	Pilleri, 1963
*U-13*	RH	KW	M	20	NR	Faust, 1966
*U-14*	RH	Charles Whitman	M	NR	NR	de Chenar, 1966; Martinius, 1983
*U-15*	RH	FL	M	56	NR	Lesniak et al., 1972
*U-16*	RH	RN	M	Birth trauma	NR	Martinius, 1983
*U-17*	RH	PL	M	4	R	Marlowe, 1992
*U-18*	RH	Case 11.3	M	27	NR	Benson, 1994
*U-19*	RH	NR	M	33	NR	Cohen et al., 1999
*U-20*	RH	MGS	M	20	R	Dimitrov et al., 1999
*U-21*	RH	Subject B/ML/SB-2046/D2	M	3 months	L	Anderson et al., 1999, 2000, 2006; Tranel et al., 2002
*U-22*	RH	Case 2	M	65	R	Mendez et al., 2000
*U-23*	RH	NR	M	14	R	Crucian et al., 2000
*U-24*	RH	NR	M	13	R	Nyffeler and Regard, 2001
*U-25*	RH	CB-2310	W	71	R	Tranel et al., 2002
*U-26*	RH	DV-1589	M	32	R	Tranel et al., 2002
*U-27*	RH	RW-1768	M	54	R	Tranel et al., 2002
*U-28*	RH	NR	M	24	R	Burns and Swerdlow, 2003
*U-29*	RH	Case 1	M	5	NR	Nakaji et al., 2003
*U-30*	RH	Case 2	M	3.5	NR	Nakaji et al., 2003
*U-31*	RH	D3	M	3	A	Anderson et al., 2006
*U-32*	RH	D4	M	Birth defect	R	Anderson et al., 2006
*U-33*	RH	PF1	M	3 days	L	Anderson et al., 2007
*U-34*	RH	2713	M	46	R	Tranel and Bechara, 2009
*U-35*	RH	NR	M	39	R	Devinsky et al., 2010
*U-36*	RH	Patient B	M	Uncertain	NR	Jonker et al., 2011
*U-37*	RH	Case 2	M	±60	R	Scarpazza et al., 2018

a *LH, left hemisphere; RH, right hemisphere*.

b *M, man; NR, not reported; W, woman*.

c *Age expressed in years unless stated otherwise*.

d *A, ambidextrous; L, left-handed; NR, not reported; R, right-handed*.

**Table 2:** Acquired sociopathy due to Bilateral (B) hemispheric damage (mania, mental retardation, and dementia excluded).

**Code**	**Identification**	**Sex^a^**	**Age at injury^b^**	**Handedness^c^**	**References**
*B-1*	Franz Binz	M	32	NR	Welt, 1888
*B-2*	Case 1	M	25	NR	van Gehuchten, 1913
*B-3*	JA	M	41	NR	Brickner, 1939, 1952
*B-4*	KM	M	16	R	Hebb and Penfield, 1940
*B-5*	JP	M	4	NR	Ackerly, 1964
*B-6*	AL	M	adulthood	NR	Goldar and Outes, 1972
*B-7*	AB	M	±50	NR	Regestein and Reich, 1978
*B-8*	EVR	M	35	R	Eslinger and Damasio, 1985
*B-9*	Case 1	M	39	R	Miller et al., 1986
*B-10*	GK	M	7 days	R	Price et al., 1990
*B-11*	SG 1208	M	71	R	Damasio et al., 1990
*B-12*	DM 1336	M	adulthood	R	Damasio et al., 1990
*B-13*	SN	M	11	NR	Williams and Mateer, 1992
*B-14*	Mrs. C	W	39	R	Ortego et al., 1993
*B-15*	MG	M	congenital	NR	Benítez et al., 1996
*B-16*	Case 1	M	21	NR	Jurado and Junqué, 2000
*B-17*	JS	M	56	R	Blair and Cipolotti, 2000
*B-18*	NR	M	36	R	Frohman et al., 2002
*B-19*	CD	M	26	NR	Cato et al., 2004
*B-20*	318	M	adulthood	R	Anderson et al., 2006
*B-21*	1106	M	adulthood	R	Anderson et al., 2006
*B-22*	1445	M	adulthood	R	Anderson et al., 2006
*B-23*	1584	M	adulthood	R	Anderson et al., 2006
*B-24*	1643	M	adulthood	R	Anderson et al., 2006
*B-25*	CL	M	14	R	Mitchell et al., 2006a,b
*B-26*	Case 1	M	36	R	Koenigs et al., 2007
*B-27*	Case 2	M	48	R	Koenigs et al., 2007
*B-28*	Case 6	M	59	R	Koenigs et al., 2007
*B-29*	HN	M	53	NR	Namiki et al., 2008
*B-30*	Patient 8	M	56	NR	Mendez et al., 2011
*B-31*	Patient A	M	adulthood	NR	Jonker et al., 2011
*B-32*	Case 2	M	congenital	NR	Trebuchon et al., 2013
*B-33*	Case 3	M	uncertain	NR	Trebuchon et al., 2013
*B-34*	NR	M	32	R	Fumagalli et al., 2015
*B-35*	NR	M	62	NR	Sartori et al., 2016
*B-36*	MH	W	4	A	Price et al., 1990
*B-37*	FL 1164	W	adulthood	R	Damasio et al., 1990
*B-38*	HS 1065	W	adulthood	R	Damasio et al., 1990
*B-39*	SAL	W	38	R	Cicerone and Tanenbaum, 1997
*B-40*	FD	W	15 months	A	Anderson et al., 2000
*B-41*	Case 1	W	35	NR	Jurado and Junqué, 2000
*B-42*	1164	W	adulthood	R	Anderson et al., 2006
*B-43*	1942	W	adulthood	R	Anderson et al., 2006
*B-44*	Case 3	W	33	R	Koenigs et al., 2007
*B-45*	Case 4	W	50	R	Koenigs et al., 2007
*B-46*	Case 5	W	53	R	Koenigs et al., 2007
*B-47*	Case 1	W	congenital	R	Trebuchon et al., 2013
*B-48*	GC	W	early developmental	R	Ibáñez et al., 2018

a *M, man; NR, not reported; W, woman*.

b *Age expressed in years unless stated otherwise*.

c *A, ambidextrous; L, left-handed; NR, not reported; R, right-handed*.

### Statistical Analysis

Because most variables of interest were categorical, they were analyzed with the χ^2^ and related statistics. A significance threshold of 0.05, two-tailed, was set for all tests. Statistical power and effect sizes were computed with Cramér's *V* and classified as small (0.10), medium (0.30) or large (0.50) following Cohen's guidelines (Cohen, 1992).

## Results

Tables 1–4 present the main results of the literature survey. Duplicate cases are also indicated in the tables. The main features leading to a diagnosis of acquired sociopathy in each case are presented in Tables 3 and 4 for the unilateral and bilateral cases, respectively. Readers may thus judge for themselves the appropriateness of the present analysis.

**Table 3:** Antisocial features in acquired sociopathy due to Unilateral Hemispheric Damage (U).

**Code**	**Features supportive of a diagnosis of sociopathy**
*U-1*	Excellent premorbid moral conduct. Arrested twice for robbery. Apathetic and indifferent to the disgrace and discomfort of imprisonment. More irritable than used to be.
*U-2*	The patient developed subtle changes in personality and displayed poor financial judgment. He began to make sexual proposals toward his 7-year-old daughter and her friends. Arrested for propositioning children in his neighborhood.
*U-3*	Honest and reliable as a worker and husband prior to brain injury. Thereafter, he became emotionally unstable, disinhibited and impulsive. Developed irresponsible behavior at work and at home. Unable to reassume regular jobs. Bankruptcy. Divorce.
*U-4*	Normal social and emotional development. Personality changes included impairment of emotional expression and in the establishment of meaningful relationships. Sexually promiscuous up to seven boyfriends at a time. Unable to hold a job for more than a few weeks despite intact operational learning. At work, poor interpersonal skills, inability to execute the required activities, and failure to learn from mistakes.
*U-5*	The subject was a college graduate who had a successful career as an advertising executive. He was involved in gambling activities. Strangled his wife and threw her body out of the window of a 13th floor apartment. He systematically arranged the crime scene in order to make it look like she committed suicide. An insanity defense was presented but the subject was convicted of manslaughter and sentenced to 7–21 years in prison.
*U-6*	Childhood marked by social isolation and aggression. The subject was convicted of murder and rape. Exhibited profound difficulties in representing the mental states of others.
*U-7*	Worked as a responsible bookkeeper before surgery. Soon thereafter, became impulsive and unpredictable. Impairments of social conduct, emotional processing, and decision-making. Discharged due to unreliability in her work attendance. Unable to hold other jobs.
*U-8*	The subject exhibited deviant sexual behavior. Convicted multiple times of sexual attacks.
*U-9*	After left temporal lobectomy, the patient developed prominent difficulties in emotional functioning and personality, marked especially by emotional lability and irritability. She was dismissed from her job due to inability to perform her duties. Disinhibition, impulsivity and perseveration were also observed during neuropsychological examination.
*U-10*	Normal developmental milestones. Parents reported uncharacteristic behavior including stealing, lying, aggression, rage, rude language, and disobedience. Lack of response toward punishment. Impulsivity and disrespect for authority. Hypersexual behavior. Arson.
*U-11*	Sentenced to 1 year in prison for a pedophilia-related offense, namely, physical assault and sexual harassment of his pubescent stepdaughter. Also exhibited depression, apathy and mild aggressive behavior. No premorbid history of sexual deviancy. A large tumor in the left frontal lobe on MRI.
*U-12*	Evaded school since an early age. Known in his birthplace as the “Original” and the “Failure.”
*U-13*	Came from a highly industrious family and worked as a baker. After suffering a traumatic occipital head injury, he developed transient loss of vision, after which he feigned severe visual loss, and became sexually promiscuous. Had numerous (heterosexual) affairs outside his marriage, often slept in the streets and did not care for his family. Alcoholic. Increasingly reported for neglect, loitering and begging. Known by his relatives as “the scoundrel of K.”
*U-14*	Killed 16 people, including his mother and wife, and wounded other 32.
*U-15*	Charged for assaulting and having incestuous intercourse with his 14-year-old daughter as well as for stripping himself naked and exhibiting his penis to two teenage girls. Caught several times by his wife in the cowshed having sex with cows and calves.
*U-16*	Complicated birth. The patient exhibited destructive play behavior and inability to adjust to peer group at kinder garden. In school, he frequently engaged in fights for minor reasons and became offensive toward women. Murdered an 8-year-old boy by beating, strangling and stabbing him after being insulted. In a psychiatric unit he became easily irritated and displayed hostile attitude toward staff members. Overall intellectual capacities were slightly below average.
*U-17*	Normal premorbid history. Aggressiveness, emotional lability and restlessness. Reacted violently with kicking, hitting and cursing at anyone who attempted to curb his bad behaviors without remorse afterward. The patient was suspended from school in the first grade for refusing to attend classes and assaulting the vice-principal as she attempted to restrain him from escaping school.
*U-18*	Attained the rank of captain in the U.S army; regarded as a strict and evenhanded officer. After the lesion, he started to exhibit tendencies toward poorly controlled hedonic acts including sexual advances. His conversation was blatant, frank, often caustic and unpleasant to those around. Fired from his last job due to verbal dysdecorum. Behavior described as impulsive and self-serving.
*U-19*	Worked for 10 years as a test driver. Married. No medical or psychiatric history prior to injury. Compulsive urge to “borrow” cars after the stroke. Arrested several times. Unable to hold a job for more than a few weeks owing to his compulsion. Over the years borrowed around 100 cars.
*U-20*	Before the injury, he had been honored with more than 10 medals in the army. After his head injury during the Vietnam War, he was demoted of rank due to ineptitude. Unable to handle jobs. Divorced three times after returning from Vietnam. His third wife was a prostitute. Arrested for shoplifting. His parents reported moodiness, sarcasm, lack of social tact, emotional blunting, remoteness of rapport, social withdrawal, inability to make and keep friends and to handle money. The probation officer had to handle his finances once he started to give large amounts of money to people on the streets.
*U-21*	Normal birth and developmental milestones. Impulsiveness and poor judgment since early childhood. Records from the school years indicated that he was disruptive in class and usually failed to turn in assignments on time despite high IQ scores. Unable to manage finances. Fired from several jobs. No realistic planning for the future. His parents described him as showing little or no worry, guilt, empathy, remorse and fear.
*U-22*	Child molestation and hypersexuality.
*U-23*	Normal development and average student at high school with age-appropriate social skills. After surgery, frequent outbursts of verbal and physical aggression. Reduced tolerance to frustration, poor control of impulses. Outbursts described as extreme and out of proportion to the triggering context.
*U-24*	Unremarkable premorbid history. Kleptomania and pathologic gambling following brain surgery.
*U-25*	Following an hemorrhagic stroke, this retired secretary underwent personality changes characterized by depression, anxiety, impulsivity, dependency, and loss of social adequacy, judgment and tact.
*U-26*	Worked as a minister and counselor prior to stroke. Thereafter, severe impairment in behavioral organization, judgment, planning, social conduct and decision-making. No longer able to maintain a job or meet the basic exigencies of everyday life.
*U-27*	Permanent and severe changes in personality and social conduct. Abulia, blunted affect and difficulty seeing tasks to fruition were reported. Unable to work.
*U-28*	The OF injury likely exacerbated a preexisting interest in pornography, sexual deviancy and pedophilia. During a neurologic exam, he solicited sexual favors from female team members. Could not refrain from acting on his pedophilia despite awareness that this behavior was inappropriate, stating that “the pleasure principle overrode” his urge restraint. Symptoms remitted after excision of the tumor.
*U-29*	Aggressive and violent behavior ranging from impulsive physical abuse of other children to an attempt to drop a brick on the head of an adult. Multiple suicide attempts. Frequently tried to ride his skateboard in the freeway. Antisocial behavior resolved after surgery.
*U-30*	Aggressive behavior composed of unprovoked screaming fits and episodic attacks of rage and violence against other children. Dismissed from 2 preschools due to his antisocial behavior.
*U-31*	Poor tolerance to frustration, emotional lability, irritability, apathy, poor judgment, social inappropriateness and impulsivity.
*U-32*	Poor tolerance to frustration, emotional lability, irritability, apathy, poor judgment, social inappropriateness and impulsivity.
*U-33*	Normal pregnancy and delivery. Difficulties in regulating the expression of disparate emotions (joy, approach, anger) in controlled laboratory settings. Absence of fear to strangers in new settings.
*U-34*	Notable changes in personality and emotional processing after right anterior temporal lobectomy. The patient exhibited marked emotional lability and irritability as reported by his wife. Impulsive and perseverative tendencies were observed during neuropsychological testing. Unable to return to his job.
*U-35*	Postsurgical hyperphagia with coprolalia, hypersexuality with *de novo* interest in pornography. Arrested at home for downloading child pornography.
*U-36*	Dramatic change of personality after car accident. Charged with multiple criminal offenses thereafter.
*U-37*	Sexually-inappropriate verbal comments during military service that never reached a physical approach. Arrested after forcing a child to touch his penis; seen masturbating close to a school. Pedophilic urges were disorganized and risky. A CT revealed a bulky meningioma over the right frontal and parietal lobes.

**Table 4:** Antisocial features in acquired sociopathy due to Bilateral Hemispheric Damage (B).

**Code**	**Features supportive of a diagnosis of sociopathy**
*B-1*	Before the accident, Binz worked as a furrier who loved telling jokes and amusing stories; benevolent, outgoing, and always in a good mood. After falling from a window 100 ft. high without losing consciousness, became quarrelsome, mean, smug and deceitful. During his hospital stay, he took pleasure in frightening and torturing the other patients, sometimes even the medical staff and his close relatives.
*B-2*	Family reported an “impossible, devastating and breaking character.” He threatened other patients with a knife and was eventually discharged from the hospital due to insubordination.
*B-3*	Worked as a stockbroker before undergoing bilateral lobectomy. Cheerful mood with occasional angry tantrums, poor judgment and lack of initiative. No appreciation of the gravity of his situation.
*B-4*	The patient exhibited childish, violent, stubborn and destructive behavior. Neighbors were terrified of him.
*B-5*	Normal development throughout infancy and early childhood. During school years, defined as disobedient, truant, and a poor helper at home. Heartily disliked by peers and lonesome. Sent to a clinic after repeated incidents of masturbation and theft at school. Stealing and running away from home were frequent.
*B-6*	Prior to the accident he was an effective employee; correct, calm and generous with good relations with friends. Following injury, became complainant, petty and aggressive. Forced his wife to have sexual intercourse in front of relatives; masturbated in public.
*B-7*	Previously a normal musician, underwent significant personality changes after a right frontal craniotomy for removal of a meningioma. Used to awakening his wife at night demanding sexual intercourse. Caught in a woodshed engaging in sexual activity with a 5-year-old girl, solicited sexual favors from a 14-year-old boy and repeatedly raped his 8-year-old nephew over a 2-year period. Displayed lack of initiative, spontaneity, emotional lability and impoverished affect.
*B-8*	Normal development. Following surgery, exhibited impaired decision-marking, drifted through several jobs being fired from all, and declared bankruptcy. Divorced twice. Indecisiveness (e.g., could take hours to decide where to dine).
*B-9*	Masturbated and attempted to have intercourse with his wife and female nurses in public.
*B-10*	Serious behavioral difficulties first identified at the age of 8. Did not respond to parental discipline, always sought gratification of his immediate needs, never developed adequate friendships, and blamed his difficulties on others. Dishonorably discharged 6 weeks after joining the Marine Corps. Hospitalized 27 times in psychiatric institutions over the ensuing 10 years; imprisoned 8 times on charges of assault, forgery, grand larceny, drug involvement and lewd behavior. Masturbated in public and was a suspect in the rape of 2 female ward patients. Showed little insight or empathy, felt victimized by others. He was given multiple diagnosis, including antisocial or borderline personality, atypical psychosis and paranoid schizophrenia.
*B-11*	Severe deficits in social conduct, judgment, and planning.
*B-12*	Severe deficits in social conduct, judgment, and planning.
*B-13*	Unremarkable premorbid history. He exhibited tantrum behavior, aggressiveness, social inappropriateness, poor judgment and a tendency to be argumentative. A lack of consideration for others and stubbornness were also reported.
*B-14*	Arrested after seducing and engaging in sexual activity with her son's girlfriend. Offered sex to other minors and requested that her son and his girlfriend engaged in sexual intercourse while she watched. No premorbid history of paraphilic behavior. Sexually inactive during the years that preceded the changes in conduct. Eventually convicted on all counts of child molestation.
*B-15*	Known as a violent man in his neighborhood. Involved in multiple criminal acts. Stole and murdered a person.
*B-16*	Sociable and respected at work prior to injury. Afterwards, engaged in thievery and other criminal activities which were performed with little or no planning: robbed a gas station without covering his face; stole caviar from the supermarket in which he was working at the time disregarding the cameras that would catch him. Except for hyperactivity, no abnormal social behavior was registered during the first years after the accident. The antisocial behavior emerged when his wife left him and under bad influences.
*B-17*	Premorbid normal behavior. The subject exhibited irritability and aggressiveness. Episodes of property damage and violence were frequent and elicited after little provocation. Recklessness regarding others personal safety. Lack of remorse. Failure to plan ahead. Unable to sustain consistent work behavior.
*B-18*	Unremarkable psychological history before the onset of aberrant sexual behavior. Approached and asked sexually explicit questions to strangers; masturbated 10–12 times a day. Reached and touched women breasts, recognizing his wrongdoings, but stating that he acted under irresistible urges.
*B-19*	Straight-A student in high school and promoted several times in the US army. After the injury, became disinhibited, jocular with a parallel decline in social and occupational functioning. Divorced three times and rejected two of his three children.
*B-20*	Behavioral changes included dampening of emotional experience, poorly modulated emotional reactions, defective decision making, especially in the social realm, impaired goal-directed behavior, and striking lack of insight.
*B-21*	Emotional blunting, poorly modulated emotional reactions, defective decision making, especially in the social realm, impaired goal-directed behavior, and striking lack of insight.
*B-22*	Behavioral changes included dampening of emotional experience, poorly modulated emotional reactions, defective decision making, especially in the social realm, impaired goal-directed behavior, and striking lack of insight.
*B-23*	Behavioral changes included dampening of emotional experience, poorly modulated emotional reactions, defective decision making, especially in the social realm, impaired goal-directed behavior, and striking lack of insight.
*B-24*	Behavioral changes included dampening of emotional experience, poorly modulated emotional reactions, defective decision making, especially in the social realm, impaired goal-directed behavior, and striking lack of insight.
*B-25*	Normal development. After his injury, he became socially isolated. His work history was sporadic, and he was routinely dismissed from jobs within weeks or even days. He was first arrested as a young adult when he sexually assaulted and murdered a middle-aged woman. The diagnosis of “post-traumatic psychopathic disorder” was made. During custody, CL was reported to behave in an abrasive, domineering and confrontational manner, lacking insight into his offense and holding an unrealistically high self-opinion. Interpersonal relations are characterized by high rates of poor behavioral controls, sexual promiscuity, grandiosity, glibness, and shallow affect. PCL-R score of 26.3 (77th percentile).
*B-26*	Striking defects in social emotion but intact intellect and normal baseline mood. Severely diminished empathy, embarrassment, guilt and impaired autonomic activity in response to emotionally charged pictures.
*B-27*	Striking defects in social emotion but intact intellect and normal baseline mood. Severely diminished empathy, embarrassment, guilt and impaired autonomic activity in response to emotionally charged pictures.
*B-28*	Striking defects in social emotion but intact intellect and normal baseline mood. Severely diminished empathy, embarrassment, guilt and impaired autonomic activity in response to emotionally charged pictures.
*B-29*	Graduated from high school and ran his own company, working as a highly skilled crane operator. After injury he became apathetic and indifferent. The subject urinated in inappropriate places and displayed anger against his family. Unable to hold finances. Signed contracts without understanding their content. Marked disturbance recognizing facial emotions.
*B-30*	Poor decision making in business; uncharacteristic sexual promiscuity with many recent affairs. Accused of indecent exposure. Poor impulse control during clinical examination.
*B-31*	The subject was prosecuted more than 20 times for multiple offenses. He exhibited aggressiveness, impulsiveness and mood changes. His last conviction was due to shoplifting.
*B-32*	Considered in school as a clever boy with no behavioral problems. During adolescence, disruptive behavior began, and the subject was expelled from school. Impulsive, hyperactive and aggressive displays of violent verbal and physical behavior. Pathological drinking and gambling. Pathological diagnosis: Taylor's dysplasia.
*B-33*	No social disturbances were reported before the onset of the seizures. His career as a football player ended due to social behavior disturbances. The subject exhibited irritability and aggressiveness toward his family, displaying repeated physical assaults which led to 10 locked ward psychiatric admissions. Showed impulsivity with episodes of self-harm, including self-induced bleeding. Expressed no regret for his actions.
*B-34*	Began to manifest pedophilic tendencies persuading 2 boys of 6 and 3 years to perform oral sex on him; displayed exhibitionism, frotteurism and voyeurism.
*B-35*	Unremarkable medical history before the onset of antisocial behaviors. Arrested after being caught enacting sexually inappropriately toward a little girl in his office. While traveling with his wife, stole postcards from exhibitors in museum shops; watched adult pornography on the web, completely worriless of being caught. Pathological crying, easy irritability, childish and obsessive-compulsive behaviors and impairments in emotion attribution, moral reasoning and abstract thinking. MRI revealed compression of the orbitofrontal cortex, optic chiasm and hypothalamus by a *clivus chordoma*.
*B-36*	Normal development until age 4, when she was struck by an automobile and remained unconscious for 48 h. Thereafter, she developed a low tolerance to frustration indicated by short-lived verbal and physical assaults against others. No sustained friendships. Poor academic performance. Sexually promiscuous. Neglected her 2.5-month-old daughter who was sent to a foster home. Her relatives lived in constant terror; they once called the police when she threatened them at knife point. Frequent outbursts against coworkers and customers in temporary jobs. Two nonplanned suicide attempts.
*B-37*	Severe deficits in social conduct, judgment, and planning.
*B-38*	Severe deficits in social conduct, judgment, and planning.
*B-39*	Graduated from college before the injury. Following discharge from hospital, her husband became concerned about her episodes of abrupt crying and laughing, rigid and obsessive behaviors during daily homemaking activities. Frequent difficulties due to indecisiveness and perplexity by minor changes in her environment, poor judgement and empathy, impulsivity, disinhibition, and self-centeredness.
*B-40*	Normal psychomotor development until age 3, when she was noted to be largely insensitive to punishment. Lied blatantly and frequently, failed to comply with school assignments and was often tardy or absent. Reprimanded for theft, intimidation, violations, destruction of property, and possession of contraband. Pregnant at the age of 18; dangerous insensitivity to infant's needs. Emotions described by relatives and caretakers as labile and poorly matched to context. Unable to articulate plans for the future.
*B-41*	Normal premorbid background. Drastic changes in conduct and personality after the lesion. Unconcerned about personal care. Drug abuse, gambling, and theft became frequent. More irritable, aggressive; did not plan for the future.
*B-42*	Emotional blunting, poorly modulated emotional reactions, defective social decision making and goal-directed behavior; striking lack of insight.
*B-43*	Emotional blunting, poorly modulated emotional reactions, defective social decision making and goal-directed behavior; striking lack of insight.
*B-44*	Striking impairment of social emotions, especially empathy, embarrassment and guilt, but normal baseline mood. Impaired autonomic reactivity to emotionally charged pictures.
*B-45*	Striking impairment of social emotions, especially empathy, embarrassment and guilt, but normal baseline mood. Impaired autonomic reactivity to emotionally charged pictures.
*B-46*	Striking impairment of social emotions, especially empathy, embarrassment and guilt, but normal baseline mood. Impaired autonomic reactivity to emotionally charged pictures.
*B-47*	Described as a “model pupil” in school. At the age of 16, she started to exhibit egocentric and impulsive behavior, frequently directing physical aggression or threats against others. Unfit to work due to seizures and social conduct problems. Served a term in prison for drug abuse and disruptive behavior. Showed no evident remorse. Pathological diagnosis: Taylor's dysplasia.
*B-48*	Disinhibition, impulsivity and disruption of social norms. Expelled from school due to impulsive behavior and recurrent aggression against peers.

### Acquired Sociopathy From Circumscribed Brain Damage

**Figure 1:**
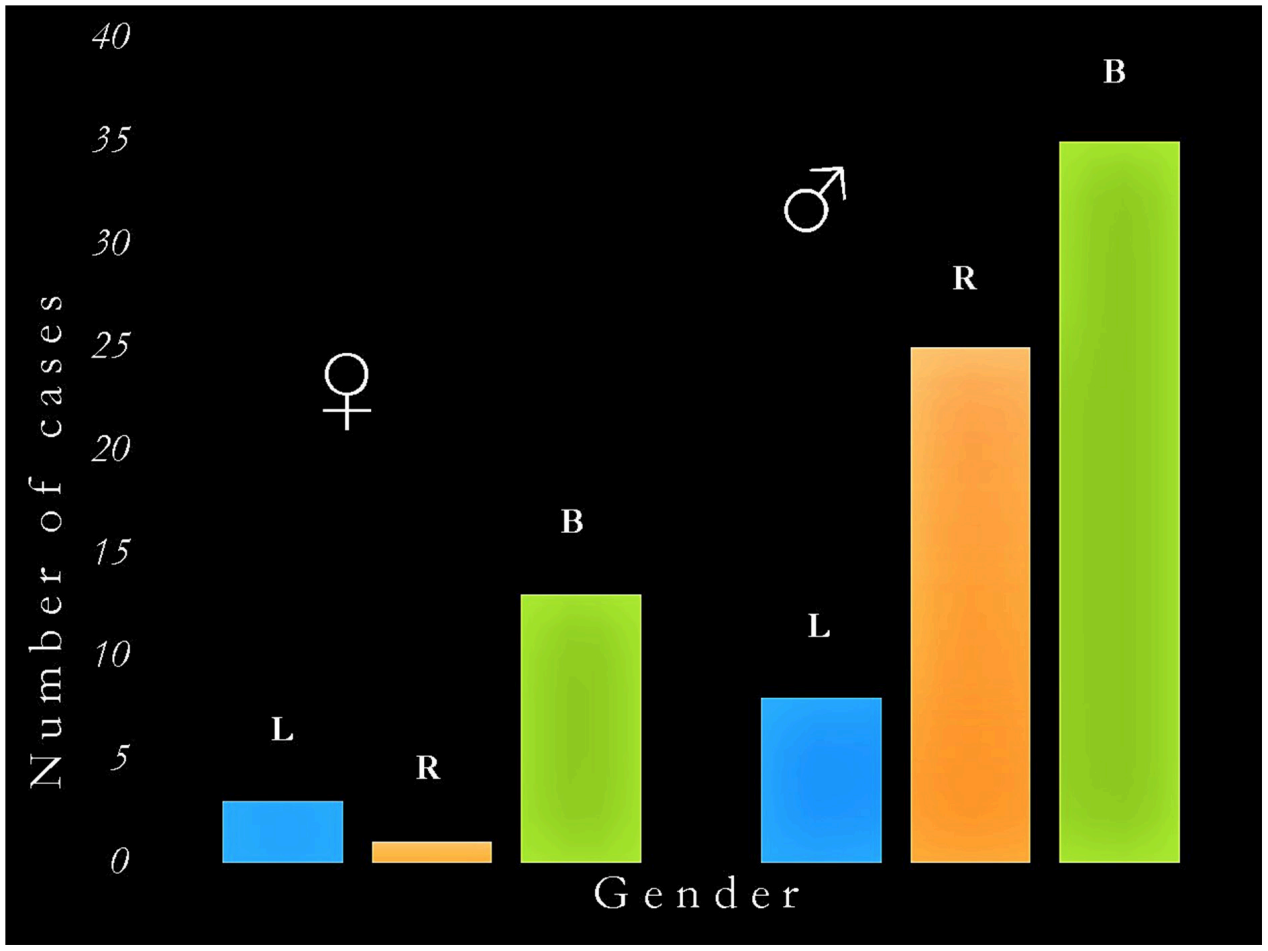
Graphic representation of the interaction between gender and hemispheric side of damage in acquired sociopathy. The critical determinants of acquired sociopathy are (a) being male, and (b) damage to the right hemisphere (either unilateral or as part of bilateral lesions). B, bilateral damage; L, left hemisphere damage; R, right hemisphere damage; ♂, men; ♀, women.

The main findings of this survey are presented in Table 5 and in Figure 1. There were 37 cases of acquired sociopathy due to unilateral hemispheric damage and 48 cases due to bilateral hemispheric damage; therefore, unilateral lesions leading to sociopathy were almost as frequent as bilateral lesions (binomial test: *p* = 0.27). There were more men than women in both the uni and the bilateral groups (binomial tests: *p* < 0.0001), as there were more cases with unilateral right hemisphere damage in the uni and the bilateral groups (binomial tests: *p* < 0.02). Information on handedness was available for 55 cases, 51 of which were right-handed, two were left-handed, and two were ambidextrous. The age of patients at the time of injury ranged from birth to 71 years (x = 28.8 ± 21.7 years), no statistically significant difference on age at injury being noted between men and women in either the uni or the bilateral groups (Mann-Whitney: *U* = 505, *p* > 0.37).

**Table 5:** Distribution of cases of acquired sociopathy according to gender and lesion laterality.

		**Women**	**Men**
Unilateral (*N* = 37)	Right Hemisphere (*N* = 26)	1	25
	Left Hemisphere (*N* = 11)	3	8
Bilateral (*N* = 48)		14	34

There was a significant correlation between gender and the uni/bilaterality of brain damage (χ^2^ = 6.1, *df* = 1, *p* < 0.04; Cramér's *V* = 0.23) as well as between gender and unilateral lesions (χ^2^ = 4.4, *df* = 1, *p* < 0.04) corresponding to medium effect sizes (Cramér's *V* = 0.35). *When only men were considered*, the number of cases with uni (*N* = 37) and bilateral (*N* = 34) damage did not significantly differ (χ^2^ = 0.13, *df* = 1, *p* > 0.72); however, the number of men with a left hemisphere injury (N = 8) was significantly lower than those with an injury of the right (*N* = 25) or of both (*N* = 34) hemispheres (χ^2^ = 16.4, *df* = 2, *p* < 0.0001). *When only women were considered*, the number of cases with bilateral damage (*N* = 14) was significantly higher than those with a unilateral (*N* = 4) lesion (χ^2^ = 5.6, *df* = 1, *p* < 0.02); moreover, there were more cases with bilateral than either with unilateral right (*N* = 1) or left (*N* = 3) damage (χ^2^ = 16.3, *df* = 2, *p* < 0.0001). Ordinal regression revealed a significant association between acquired sociopathy and right hemisphere damage in men (OR = 10.3, *p* < 0.02). No equivalent associations were seen for women regardless of the laterality or bilaterality of damage (OR = 1.1, *p* > 0.90). The inclusion of handedness and age at which the brain injury was acquired in the regression model did not qualitatively change these results.

Finally, it may seem surprising not to find the case of Phineas Gage in the Tables. The reason for this is the dispute regarding the bilaterality (Damasio et al., 1994) or the unilaterality (Ratiu and Talos, 2004) of Gage's injury, which remains speculative because no postmortem exam was performed on Gage's brain. In addition, the details of Gage's case have often been embellished, distorted, or inferred beyond what is justified by the first-hand reports by Harlow and Bigelow (Macmillan, 2008).

## Discussion

The present survey came to some intriguing and unexpected results which may be thus summarized: (i) unilateral lesions are almost as frequent as bilateral lesions leading to acquired sociopathy; (ii) there are relatively few well-documented cases of acquired sociopathy in the medical literature, especially when we consider the length of the historical record; (iii) acquired sociopathy was significantly more common in men after an injury of the right or of both cerebral hemispheres; (iv) in most women who developed acquired sociopathy the lesions affected both cerebral hemispheres; and (v) men outnumbered women in both the unilateral and bilateral groups.

As stated in the Material and Methods section, the point of departure of the present analyses was clinical, not anatomical. The logical next step is to follow the reverse course of case selection beginning with the collection of cases with lesions of the right and the left frontotemporoinsular cortices, and investigate their clinical manifestations according to gender and lesion laterality. These two approaches are not comparable, and the anatomical approach may lead to quite different conclusions.

### Gaps in Need of Further Research

Owing to the limitations of the material herein surveyed, the present review should be considered cautiously. These limitations have to do with missing data in several reports, including the handedness of participants, the age at which the brain damage occurred, and when the manifestations of sociopathy were first noted; however, the most important gap is the fact that the anatomical descriptions are often too vague to allow in-depth clinicoanatomical deductions.

Besides the aforementioned gaps in knowledge, the present survey reveals that the number of well-documented cases of acquired sociopathy is surprisingly small, especially in view of the popularity that it has long enjoyed both among scholars and in the lay media (de Oliveira-Souza and Moll, 2019). This partly unfounded popularity has given breadth to the assumption that we know more about the cerebral underpinnings of acquired sociopathy than we actually do. Indeed, one of the main purposes of the present work was to gather these gaps in knowledge in a single communication. There is a pressing need to revive the clinicoanatomical tradition of studying single cases or case series with modern behavioral and neuroimaging methods. Despite its limitations, the postmortem exam remains the gold standard for increasing our knowledge of neural structures and pathways (Goetz, 2010). This assertion is bolstered by the exemplary studies that make up the body of the present survey (e.g., Dimitrov et al., 1999; Cato et al., 2004; Eslinger et al., 2004; Anderson et al., 2007).

### Gender and Lesion Asymmetries in Acquired Sociopathy

Acquired sociopathy was significantly more common in men after an injury of the right or of both cerebral hemispheres. A corollary of this finding is that the left hemisphere in men who develop acquired sociopathy due to bi-hemispheric injuries probably plays a minor, if any, role in the genesis of the antisocial personality changes, a finding that is in line with Tranel et al. observations (see Introduction). In most women with acquired sociopathy, in contrast, injuries of both hemispheres were significantly more common than lesions of either hemisphere alone, a finding that is partially in line with Tranel et al. observations.

Despite the wealth of research on cerebral asymmetries and on the differences between the brains of men and women (Cahill, 2006), the neural underpinnings of the *interactions* between asymmetry and gender have been less studied. The gender and lateral asymmetries in acquired sociopathy revealed by the present survey provide converging evidence that the neural organization of sociomoral conduct in women has a more symmetrical organization in the cerebral hemispheres than in men, in whom the right hemisphere seems to play a dominant role. This conclusion, however provisional, is supported by the broader issue of gender differences in sociopathy (Moffitt et al., 2004) and by the cerebral organization of moral cognition and behavior in men and women (Dulebohn et al., 2016).

In most of the cases herein reviewed, the lesions were in a position to either directly destroy or impinge upon the uncinate fascicle (UF), the ventral amygdalofugal pathway, and the medial forebrain bundle. The UF is a hook-shaped association tract that interconnects the anterior temporal lobe, the insula, and the orbitofrontal and ventromedial prefrontal cortices (Ebeling and von Cramon, 1992). It is composed of five smaller, partially overlapping, fascicles with different origins, trajectories, and terminations (Hau et al., 2017); a remarkable rightward asymmetry of the UF, both in volume and number of fibers, has consistently been noted by researchers (Highley et al., 2002). The UF integrates a collection of cortical areas which play an essential role in the regulation of higher-order social behavior, as shown in extreme form by the human Klüver-Bucy syndrome (Hayman et al., 1998). This functional unity is also profusely interconnected with the amygdala and the basal forebrain bidirectional fiber systems that are responsible for the basic drives and the regulation of the internal milieu of the organism (Livingston and Escobar, 1971), thus allowing the reciprocal interaction between complex social and fundamental organic behaviors (Mogenson and Yang, 1991). For the most part, the frontotemporoinsular cortices fall within the projection fields of the UF, the ventral amygdalofugal pathway (Kamali et al., 2016), the stria terminalis (Baydin et al., 2017), and the inferomedial leaflet of the medial forebrain bundle (Edlow et al., 2016). These tracts funnel through the temporal stem toward the temporal pole and amygdala, a critical region of the white matter in which relatively small injuries may produce major disturbances in moral conduct and social behavior (Kling et al., 1993). The right UF has been shown to be reduced in individuals with psychopathy (Sobhani et al., 2015), but the integrity of other white matter pathways may be disrupted as well (Waller et al., 2017). Thus, there are good reasons to suppose that the UF and neighboring tracts also play a critical role in the manifestations of acquired sociopathy (van Horn et al., 2012).

### The Male Preponderance in Acquired Sociopathy: Fact or Artifact?

When we began this project, we had no a priori expectations regarding the relative prevalence of men to women in acquired sociopathy. Neither do we now have a definitive explanation for the higher male-to-female preponderance either in the unilateral (7.2:1) or bilateral (2.3:1) groups. The most parsimonious explanation is provided by epidemiological studies, which indicate that the male preponderance of the present survey is a particular instance of a general phenomenon shared by common neurological illnesses such as stroke, head injury, and epilepsy (Braun et al., 2002). For example, men are three times more likely to suffer a traumatic brain injury than women (Gardner and Zafonte, 2018). The unequal proportion of genders in the literature should not divert us from the possibility that, as far as can be judged from the available cases, the occurrence of acquired sociopathy in injuries of the cerebral hemispheres seems to be strongly modulated by gender and lesion laterality. This hypothesis concurs with a growing body of evidence indicating that gender differences in various aspects of normal social and antisocial behavior mainly reflect neurobiological mechanisms rather than cultural modeling.

## Final Remarks

Over a century ago, van Gehuchten (1913) and Agostini (1914) entertained the possibility that the laterality of a lesion in the frontal lobes played a critical role in the determination of acquired sociopathy. At that time, and for the ensuing seven decades, the means to test this possibility relied on postmortem exams, which usually reflect the final stages of the pathological process and often lay distant in time from the clinical and behavioral manifestations of interest. Such drawbacks can now be circumvented by the use of current neuroimaging techniques in the assessment of social cognition (Duclos et al., 2018). The differential neural underpinnings of social cognition between men and women should systematically be considered in the design of experiments on social cognition and behavior. They may be especially valuable to neurosurgeons, and to functional neurosurgeons in particular, in the planning of interventions for tumor resections as well as the choice of the best targets for therapeutic neuromodulation (Darby and Pascual-Leone, 2017). A more precise understanding of the neurobiology of acquired and developmental sociopathy may be closer than ever before.

## Author Contributions

RdO-S, TP, JG, and JM contributed to the conception and design of the study. RdO-S and TP collected and organized the database. RdO-S performed the statistical analysis and wrote the first drafts of the manuscript. All authors contributed to manuscript revision, read, and approved the submitted version.

### Conflict of Interest Statement

The authors declare that the research was conducted in the absence of any commercial or financial relationships that could be construed as a potential conflict of interest.
